# Nonadditive and Asymmetric Allelic Expression of Growth Hormone in Hybrid Tilapia

**DOI:** 10.3389/fgene.2019.00961

**Published:** 2019-10-15

**Authors:** Huan Zhong, Xiaojin Zhang, Qian Xu, Jinpeng Yan, Zhuojun Han, Huifang Zheng, Jun Xiao, Zhanyang Tang, Fenghua Wang, Yongju Luo, Yi Zhou

**Affiliations:** ^1^Tilapia Genetics and Breeding Center, Guangxi Academy of Fishery Sciences, Nanning, China; ^2^National Demonstration Center for Experimental Fisheries Science Education, Shanghai Ocean University, Shanghai, China; ^3^Department of Cell Biology, School of Life Sciences, Central South University, Changsha, China; ^4^College of Animal Science and Technology, Guangxi University, Nanning, China; ^5^Sports Biochemistry Laboratory, Institute of Physical Education, Xinjiang Normal University, Urumqi, China

**Keywords:** growth hormone, tilapia, hybrid, heterosis, nonadditive expression, allele specific expression

## Abstract

Hybridization is a common breeding technique that can improve germplasm through heterosis in aquaculture. However, the regulation of key gene expression, including the details of transcriptional level changes at the beginning of hybridization events, remains largely undefined, especially in teleosts. In this study, by interspecies crossing between two pure lines of Nile tilapia and blue tilapia, we obtained a hybrid tilapia population as a model to elucidate heterosis, and we traced the molecular outcomes of growth hormone (GH) expression and allele-specific expression (ASE) in hybrids. The hybrids display growth vigor compared to their parents in the 120-day growth trial. GH mRNA expression was uniquely expressed in the pituitary. Higher GH expression was found in the hybrid than the midparent value, in both males and females, showing a nonadditive pattern. We identified four single-nucleotide polymorphism sites between Nile tilapia and blue tilapia. Subsequently, by pyrosequencing, we found asymmetric allelic expression in hybrids with higher maternal allelic transcript ratios in both males and females. Fasting significantly increased GH expression in hybrids, but asymmetric allelic expression was not affected by feeding or fasting conditions. Finally, we identified *cis* and *trans* effects *via* overall expression and ASE values in the hybrid, which showed that the *cis* and *trans* effects promoted the expression of maternal subgenome in the hybrid, contributing to the expression superiority of GH in hybrid tilapia. Taken together, the results of our study first illustrated the concept of GH expression superiority and its formation mechanism in hybrid fish with growth vigor.

## Introduction

Interspecific hybridization is common in plants but occurs less in animals, especially in vertebrates. However, several interspecific hybrid fishes have been found in natural rivers (Deines et al., 2014) and lakes (Taylor et al., 2002; Smith et al., 2003). These successively formed hybrids indicate that interspecific hybridization is the driving force in speciation. Fusion of two genomes may lead to novel phenotypic variation that guarantees survival in competition with parents (Mallet, 2007). These phenotypes could be fast growth (Sun et al., 2016) or enhanced immune function (Liu et al., 2004). Vigor phenotypes are attributed to heterosis. However, heterosis is uncertain, depending on parents. For example, Nile tilapia (*Oreochromis niloticus*, female) × blue tilapia (*Oreochromis aureus*, male) hybrid shows a faster growth rate when compared to its parents, but no such growth vigor could be found in blue tilapia (male) × Nile tilapia (female) hybrid (Pruginin et al., 1975). Thus, understanding the molecular mechanisms of heterosis in hybrids is crucial for hybridization speciation and cross-breeding.

Fusion of two distant in F_1_ hybrids leading to genomic instability was described by McClintock (1984) as “genome shock.” These genetic changes include DNA fragment loss (Han et al., 2003), modification of DNA methylation levels (Abid et al., 2011), and transposon activities (Kidwell and Lisch, 1998) affecting phenotypes of hybrids. In addition, recent studies represent novel changes, such as “gene expression shock” or “transcriptome shock,” in hybrid plants (Hegarty et al., 2006), which is defined as extensive changes to patterns of parental gene expression. Clearly, gene expression patterns in hybrids are associated with heterosis, which makes hybrid speciation successful. Overall expression of homologues in hybrids is related to unique phenotypes, such as fast growth (Sun et al., 2016) and strong resistance to pathogens (Zhang et al., 2006). By comparison with its parents, gene expression in hybrids could be classified as additive and nonadditive gene expression (Ren et al., 2016; Zhu et al., 2017b). Nonadditive gene expression has been regarded as a specific expression pattern for heterosis (Li et al., 2015). In hybrid, nonadditive gene expression could be confirmed when its expression is not equal to the midparent value (MPV). In addition to the overall expression pattern, allele-specific expression (ASE) has been suggested as another mechanism of heterosis (Shao et al., 2019). ASE is often associated with sequence variation in regulatory regions from subgenomes in hybrids (Cheng et al., 2016). Evidence in plants has demonstrated that allelic imbalance is pervasive and leads to phenotypic variations (Zhang et al., 2018).

Gene expression changes of overall expression and ASE in hybrid are mediated by *cis*- and/or *trans*-regulatory changes (Wittkopp et al., 2004). Interspecific F_1_ hybrids are particularly well suited for identifying *cis*- and/or *trans*-regulatory changes by analyzing their expression characteristics (Coolon and Wittkopp, 2013). *cis*-Regulatory elements located on transcribed regulatory regions participate in regulating transcriptional activity and stabilizing mRNAs (Wittkopp and Kalay, 2012). In contrast, *trans*-regulatory elements, such as transcription factors and noncoding RNAs, affect transcriptional activities by intermolecular interaction (Sassone-Corsi and Borrelli, 1986). These regulatory changes in hybrids have been demonstrated in yeast (*Saccharomyces cerevisiae*) (Emerson et al., 2010), maize (*Zea mays* L.) (Guo et al., 2008), *Drosophila* (Landry et al., 2005), and mouse (*Mus musculus*) (Goncalves et al., 2012) models. The majority of regulatory changes were dominated by *cis* effects based on these results in hybrids, while environmental conditions affect gene expression in a *trans*-regulatory manner. Notably, recent finding has shown that *cis*- and *trans*-regulatory elements are coevolved and influence phenotypes of hybrids collectively *via* regulating gene expression (Chen, 2013).

The Nile tilapia (female) × blue tilapia (male) hybrid is a crossbreed that has been found in natural rivers and lakes (Deines et al., 2014), representing a primitive state of hybrid speciation. Moreover, this breeding line shows heterosis for growth rate and disease resistance (Wu et al., 2011; Lai et al., 2012); thus, the hybrid line has been widely cultured around the world in the aquaculture industry. Although studies in plants and *Drosophila* have explained the expression changes in hybrids affecting heterosis, direct information about heterosis in vertebrates, especially in a naturally formed hybrid fish line, is less well known. To illustrate the successful survival in nature of a newly formed crossbreed and its growth heterosis, we generated a hybrid line using two pure strains of Nile tilapia and blue tilapia. This hybrid line is genetically tractable for the present study. In teleosts, growth hormone (GH)/insulinlike growth factor (IGF) axis is crucial for regulating somatic growth (Reinecke et al., 2005). GH is predominately secreted in pituitary and stimulates IGF-1 expression to promote somatic growth (Vijayakumar et al., 2011). Thus, GH is an important upstream gene for promoting growth appearance. In this study, we focused on the growth vigor of hybrid tilapia and the expression patterns of GH. Our analysis suggested that the expression characteristics of GH may have distinct implications in the heterosis of hybrids.

## Materials and Methods

### Fish Materials and Reared Conditions

Nile tilapia, blue tilapia, and hybrids were obtained in National Tilapia Breeding Fields of Nanning from Guangxi Academy of Fishery Sciences (Nanning, China). The original Nile tilapia and blue tilapia were introduced from the Freshwater Fisheries Research Center of the Chinese Academy of Fishery Sciences, Wuxi, China. After nine rounds of self-mating, the 10th generations of Nile tilapia and blue tilapia were generated as pure lines with clear genetic backgrounds. Subsequently, hybridization was performed using 20 female Nile tilapias and 10 male blue tilapias by rearing in one pond (5.0 × 2.0 × 1.5 m). Meanwhile, self-mating of Nile tilapia and blue tilapia was performed. After 15 days, 200 fry of each combination was collected, and the different fry were reared in three separate ponds (10.0 × 5.0 × 2.0 m). The fish grew to adults and were used in the present analyses. The present experiments were approved by the Animal Research and Ethics Committees of the Guangxi Academy of Fishery Sciences.

### Growth Trial

A growth trial was performed to evaluate the growth performance of the hybrid and its parents. A 150-day growth period is the most common culture period in South China, and the fastest growth period is between 1 and 5 months for tilapia. Thus, we used a 120-day trial from 1- to 5-month-old fish to evaluate the growth differences among the different fishes. Because males grow faster than females, the farming tilapias are usually all males to yield maximum production. In addition, hybrid tilapia has high male rate (up to 90%). We only measured the growth appearance of male in the present study. The body weights of Nile tilapia, blue tilapia, and hybrids (20 males of each fish) were measured at the beginning of the trial when the fry was 1 month old. The experimental Nile tilapia, blue tilapia, and hybrids were reared in three adjacent ponds. All of the fish were fed commercial diets with 2% body weight per day, and the conditions (including pH, dissolved oxygen, NH_3_–N) of the ponds were assayed each week. The water conditions of the experimental ponds were as follows: pH 7.20 to 8.00; dissolved oxygen 6.50 to 8.00 mg/L; and NH_3_–N 0.05 to 0.20 mg/L. These similar conditions excluded environmental effects on growth performance. After 120 days at the end of the trial (the fish was 5 months old), 20 males of each fish were randomly selected and measured. The specific growth rate (SGR) was calculated using the following formula: SGR = 100 × (Ln final weight - Ln initial body weight)/trial days according to the previous study (Ng et al., 2000).

### Evaluation of Overall GH Expression

Total RNA in adult Nile tilapia, blue tilapia, and hybrid was extracted using TRIzol reagent (Takara, Japan). The quality and quantity of the extracted RNA were determined by 1% agarose gel and SmartSpec^™^ Plus Spectrophotometer (Bio-Rad, USA) based on 260- and 280-nm absorbance, respectively. One microgram of total RNA was converted to cDNA by the PrimeScript^™^ II 1st Strand cDNA Synthesis Kit (Takara, Japan). Total RNA was isolated using TRIzol reagent (Takara, Japan), and cDNA was synthesized using the PrimeScript^™^ RT reagent Kit with gDNA Eraser (Takara, Japan). A pair of GH primers for expression analysis was designed by Primer Express v3.0 (Applied Biosystems, USA) (**Table 1**).

**Table 1:** Primers used in the present study.

Primer names	Sequences (5′ to 3′)	Position	Annealing temperature	Amplicon size	Usage
GH-forward	CTGAGCCGCAAACAGAGCC	-26 to -8	60°C	777 bp	cDNA cloning
GH-reverse	AAAACTTCTGATGTCACGATTACCA	727–751			
rt-GH-forward	ATCAGGGCCAATCAGGATGA	409–428	60°C	150 bp	DNA sequencing, RT-PCR and qPCR
rt-GH-reverse	GTGCATGTCCTTCTTGAAGCAA	537–558			
rt-β-actin-forward	CCACAGCCGAGAGGGAAAT	605–623	60°C	78 bp	RT-PCR and qPCR
rt-β-actin- reverse	CCATCTCCTGCTCGAAGTC	664–682			
pyr-GH-forward	GAAGCAGAGAATTATCCTGACAC	427–449	60°C	113 bp	PCR before pyrosequencing
pyr-GH-reverse	CAAGCCAGCAATTCATAAGTT	519–539			
pyr-GH	ACTATTATCAAAGTCTGGGAG	479–499	Not applicable	Not applicable	Pyrosequencing

Position indicates the primer locations in genes from start codon (ATG).

The muscle, pituitary, heart, spleen, intestine, brain, ovary, testis, head-kidney, and liver were used to analyze the tissue distribution of GH mRNA expression using reverse transcriptase–polymerase chain reaction (RT-PCR). The PCR conditions were 94°C for 5 min for initial predenaturation followed by 30 cycles of 94°C for 30 s, 60°C for 30 s, 72°C for 45 s, and a final extension at 72°C for 10 min. The PCR results were analyzed using 1.2% agarose gels.

To analyze the overall expression of GH in hybrid and its parents, two stages including the 5-month-old fish and 2-year-old fish were collected for quantitative PCR (qPCR). For each stage, pituitaries from Nile tilapia (five females and five males), blue tilapia (five females and five males), and hybrids (five females and five males) were collected for comparison expression analysis. After being anesthetized and sacrificed, the pituitaries were collected for RNA isolation, cDNA preparation, and qPCR. The primers are shown in **Table 1**, and β-actin was used to normalize the expression levels of GH in the present study. TB Green^™^ Fast qPCR Mix (Takara, Japan) was used for qPCR analysis according to the manufacturer’s instructions. The qPCR reaction was performed on a PikoReal 96 Real-Time PCR system (Thermo Fisher Scientific, USA). The PCR conditions were 95°C for 7 min followed by 40 cycles of 95°C for 5 s and 60°C for 30 s. Melting curve analysis was performed to identify the specific amplification. Relative expression levels of GH were calculated using the delta delta Ct method (Livak and Schmittgen, 2001).

### Single-Nucleotide Polymorphisms Between Nile Tilapia and Blue Tilapia

First, cDNA libraries from pituitaries in three Nile tilapias and three blue tilapias were used to identify single-nucleotide polymorphisms (SNPs) between Nile tilapia and blue tilapia. The primers for GH in tilapia were designed based on the genome (http://asia.ensembl.org/Oreochromis_niloticus/Info/Index, Ensembl version Orenil1.0, accession no. ENSONIG00000009191) of Nile tilapia (**Table 1**) using Primer Premier 5.0 software. The PCR was performed at 94°C for 5 min for initial predenaturation followed by 30 cycles of 94°C for 30 s, 60°C for 30 s, and 72°C for 45 s, and a final extension at 72°C for 10 min. The PCR products were isolated by 1.2% agarose gels and sequenced by Shanghai Sangon Biotechnology Co. Ltd. (Shanghai, China). After SNPs were identified between Nile tilapia and blue tilapia using cDNA, DNA libraries (including six Nile tilapias, six blue tilapias, and six hybrids) were further used to confirm the SNP loci. The genomic DNAs were extracted using the TaKaRa MiniBEST Whole Blood Genomic DNA Extraction Kit (Takara, Dalian, China) from blood samples according to the manufacturer’s instructions. The amplification condition was the same with PCR conditions of cDNA sequencing. The sequences were aligned by ClustalW2 (https://www.ebi.ac.uk/Tools/msa/clustalw2/), and SNPs between Nile tilapia and blue tilapia were identified.

### Allelic Expression Frequencies in Hybrid Tilapia

Pyrosequencing analysis was performed based on the C/G alleles from Nile tilapia (C) and blue tilapia (G) as the results of GH allele identification. The pyrosequencing experiments were designed and performed by Shanghai Sangon Biotechnology Co. Ltd. (Shanghai, China). Twelve pituitary cDNA samples from hybrids (six adult females and six adult males, 2-year-old fish) were included in the analysis. First, the GH fragments were amplified and extracted using 1.2% agarose gels. The purified PCR products were pyrosequenced on a PyroMark Q96 ID instrument (Qiagen, USA) by Shanghai Sangon Biotechnology Co. Ltd. (Shanghai, China). Pyrosequencing primers are shown in **Table 1**.

### Conditional Expressions of GH Alleles in Hybrid

Different feeding conditions were performed to determine whether the specific allele expression is dependent on different conditions. For feeding conditions, 10 juvenile hybrid individuals (1 month old, 6.34 ± 1.53 g) were randomly divided into two groups: feeding group and fasting group (n = 5). The hybrids were cultured in 2,000-L acrylic containers and fed commercial feed (2% body weight). After 7 days of adaptation, the feeding group was fed with 2% body weight feed. The fasting group was not fed until 48 h after the beginning of the experiment. The pituitaries were collected 48 h after the beginning of the experiment. All of the pituitaries were analyzed by qPCR and pyrosequencing for conditional expressions of GH alleles in the hybrid.

### *Cis and Trans* Effects in Hybrid Tilapia

The GH *cis* and *trans* effects were identified by overall expression and ASE according to a previous study (Shi et al., 2012). We used the results of overall expression data and pyrosequencing analysis from the adult tilapia for this analysis. First, the gene expression divergence (A) between parents was calculated by log_2_(NN/BB), and *cis* effects were evaluated by log_2_(allele of N in F_1_/allele of B in F_1_) (**Figure 6A**). The *trans* effects were determined by (A - B). The enhancing *cis* and *trans* interactions were identified as [{B > 0 and (A - B) > 0} or {B < 0 and (A - B) < 0}], showing the same direction of the regulation. The compensating *cis* and *trans* interactions were identified as [{B > 0 and (A - B) < 0} or {B < 0 and (A - B) > 0}], showing the opposite direction of the regulation.

### Statistical Analysis

The expression levels were presented using mean values ± standard deviation. For pairwise comparisons, paired *t* tests were employed. For multigroup comparisons, significant differences were confirmed by one-way analysis of variance with Tukey test for multiple comparisons. Once variances were not homogeneous, a log_10_ transform was performed before analysis. *P* < 0.05 was set as a significant difference. SPSS 16.0 was employed to conduct statistical analysis.

## Results

### Distant Hybridization and Growth Performance

**Figure 1:**
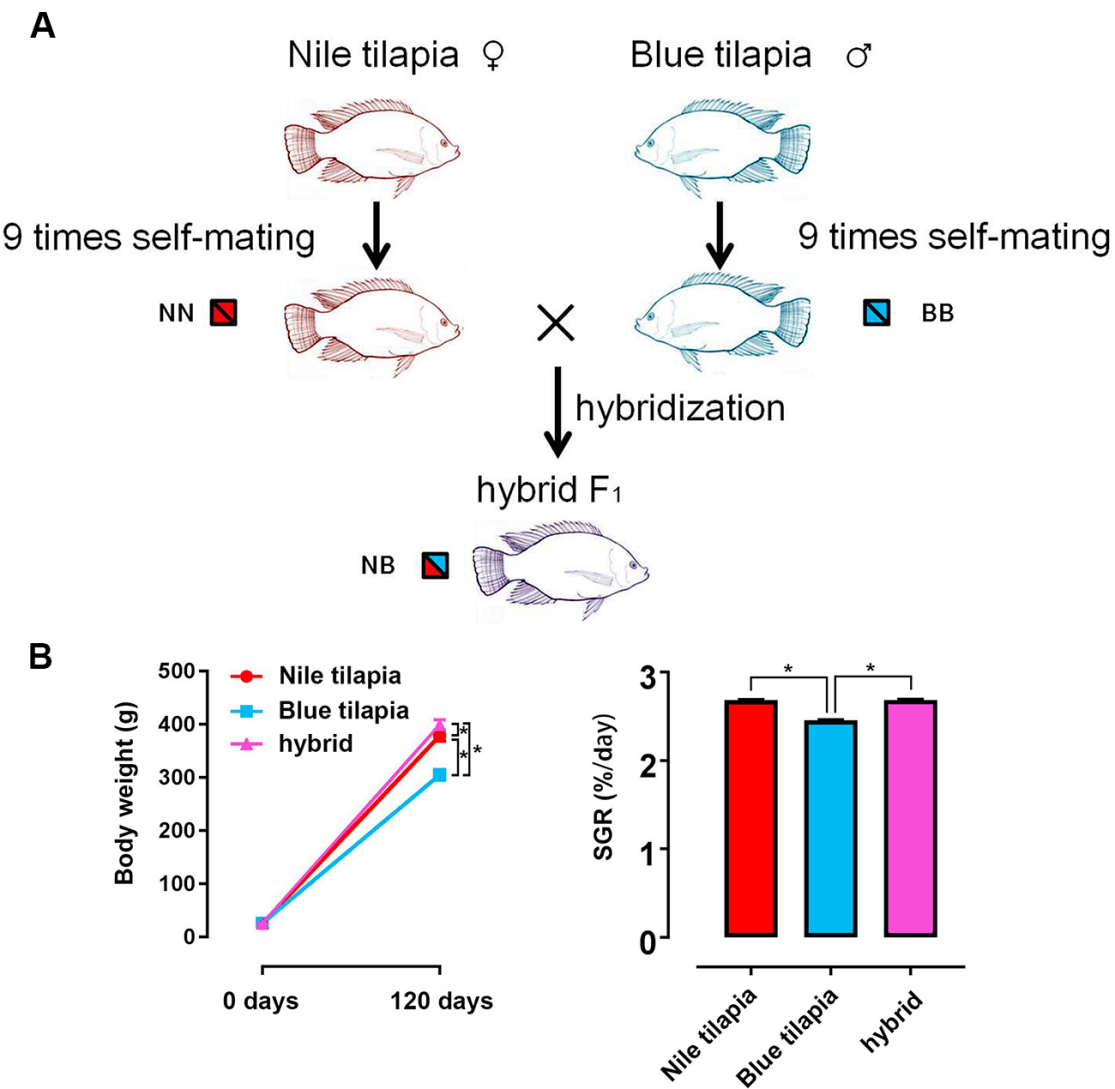
Generation of hybrid tilapia and growth performance. **(A)** The parents of the hybrid (Nile tilapia and blue tilapia) were obtained nine times after self-mating to guarantee a pure genetic background. Then, hybrid tilapia was obtained by Nile tilapia and blue tilapia crossing. The genotypes of Nile tilapia, blue tilapia, and hybrid were represented as NN, BB and NB, respectively. **(B)** Body weight and SGR comparison among Nile tilapia, blue tilapia, and hybrid. Data are presented as the mean ± SEM (n = 20). Asterisks indicate significant differences between groups (*P* < 0.05).

The hybrid tilapia was generated by interspecies crossing between Nile tilapia (female) and blue tilapia (male). Both parents were genetically purified for 10 generations by nine times self-mating. Thus, the pure genotypes of Nile tilapia and blue tilapia guaranteed a clear and traceable hybrid line for the following study (**Figure 1A**). In total, ∼5,000 hybrid fry were collected by crossing between 20 female Nile tilapias and 10 male blue tilapias. Nile tilapia and blue tilapia selfing offspring were obtained and reared for the control. The subsequent 120-day feeding trial indicated growth vigor in the hybrid. No significant differences were found at the beginning of the growth trial. However, the final weight was significantly higher (*P* < 0.05) in the hybrid (399.31 ± 9.61 g) than in the Nile tilapia (378.95 ± 9.99 g) and blue tilapia (305.25 ± 10.40 g). The growth rate was significantly faster (*P* < 0.05) in the hybrid (1,445.16% ± 36.02%) compared to its parent (1,367.96% ± 44.58% for Nile tilapia; 1,067.40% ± 47.23% for blue tilapia). There was generally no significant difference in SGR between Nile tilapia and hybrid, but blue tilapia had the lowest SGR in the three tilapias (**Figure 1B**).

### Nonadditive Expression of GH in Hybrid

**Figure 2:**
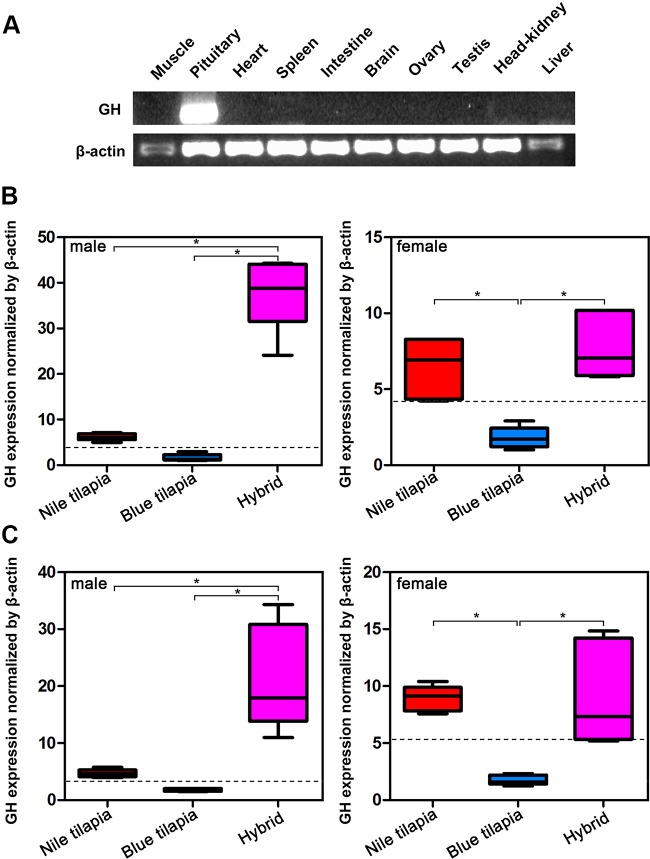
Overall expression of GH in hybrid tilapia. **(A)** Tissue distribution analysis of GH mRNA expression by RT-PCR. **(B)** Comparison of GH mRNA expression in pituitary from 5-month-old Nile tilapia, blue tilapia, and hybrid by qPCR. **(C)** Comparison of GH mRNA expression in pituitary from 2-year-old Nile tilapia, blue tilapia, and hybrid by qPCR. Data are presented as the mean ± SEM (n = 5). Asterisks indicate significant differences between groups (*P* < 0.05). Dashed lines indicate MPV calculated from the average value of Nile tilapia and blue tilapia.

Analysis of GH mRNA expression in different tissues indicated that GH was specifically expressed in the pituitary, that is, only the pituitary expressed GH mRNA (**Figure 2A**). Thus, we further compared the mRNA expression of hybrid and its parents in the pituitary. The expression changes of GH in pituitary were similar in the tilapias both at 5-month-old and 2-year-old stages (**Figures 2B, C**). In males, the highest GH mRNA expression was observed in the hybrid, while Nile tilapia and blue tilapia had significant lower (*P* < 0.05) GH mRNA expression in the pituitary. In females, both Nile tilapia and hybrids had higher GH mRNA expression compared to blue tilapia. No significant differences were found between female Nile tilapia and female hybrids. We also analyzed the expression between the hybrid and average parental values (MPV). The GH mRNA expression levels were higher in the hybrid than the MPV, both in males and females, indicating a nonadditive expression pattern of GH in the hybrid. Furthermore, the GH expression in the hybrid was classified as predominant as a subcategory in the nonadditive expression pattern, representing higher expression levels than the MPV (**Figures 2B, C**).

### ASE of GH in Hybrid

**Figure 3:**
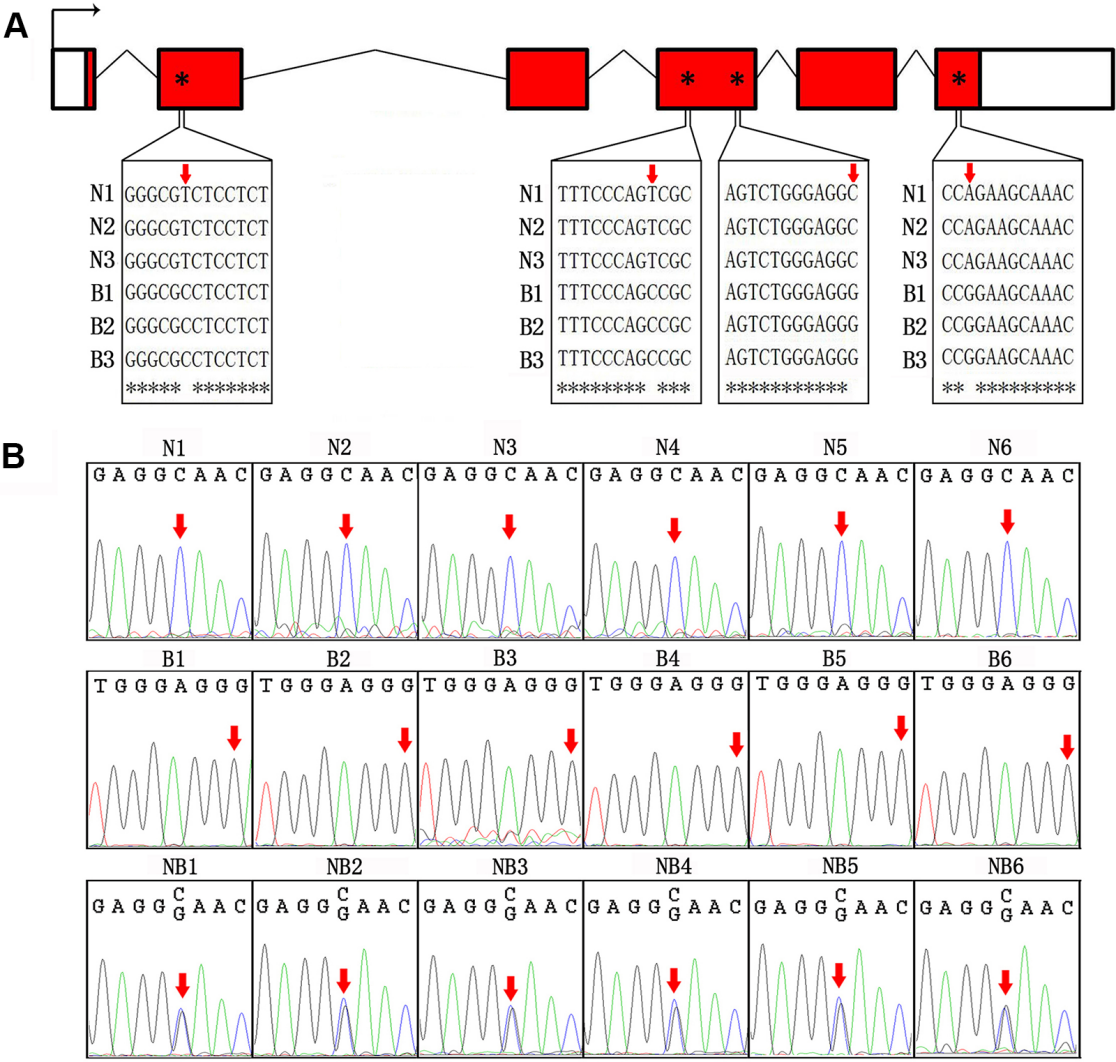
Identification of SNP between Nile tilapia and blue tilapia. **(A)** Four SNPs were identified from the cDNA sequences of Nile tilapia and blue tilapia by alignment using ClustalW. Bent arrow represents the transcription start site. Empty boxes represent the 5′ and 3′ UTRs. Red boxes represent exons. Broken lines indicate introns. Red arrows and asterisks indicate the SNP sites. **(B)** Validation of the third SNP site (nucleotide 501) from the DNA sequences by Sanger sequencing in Nile tilapia (N1–N6), blue tilapia (B1–B6), and hybrid (NB1–NB6). Red arrows indicate the SNP sites.

We first sequenced cDNA of GH from Nile tilapia and blue tilapia to identify SNPs in the two species. In the coding sequences, four SNP sites (nucleotides 53, 327, 501, and 603) were identified as specific in Nile tilapia and blue tilapia (**Figure 3A**, **Supplementary Data 1**). Furthermore, we used Sanger sequencing to validate the genotypes in Nile tilapia, blue tilapia, and hybrids from genomic DNA based on the third SNP site (nucleotide 501). Nile tilapia and blue tilapia showed specific genotypes (C and G, respectively), while the hybrid had both C and G at this site, indicating a heterozygous genotype (**Figure 3B**, **Supplementary Data 2**).

**Figure 4:**
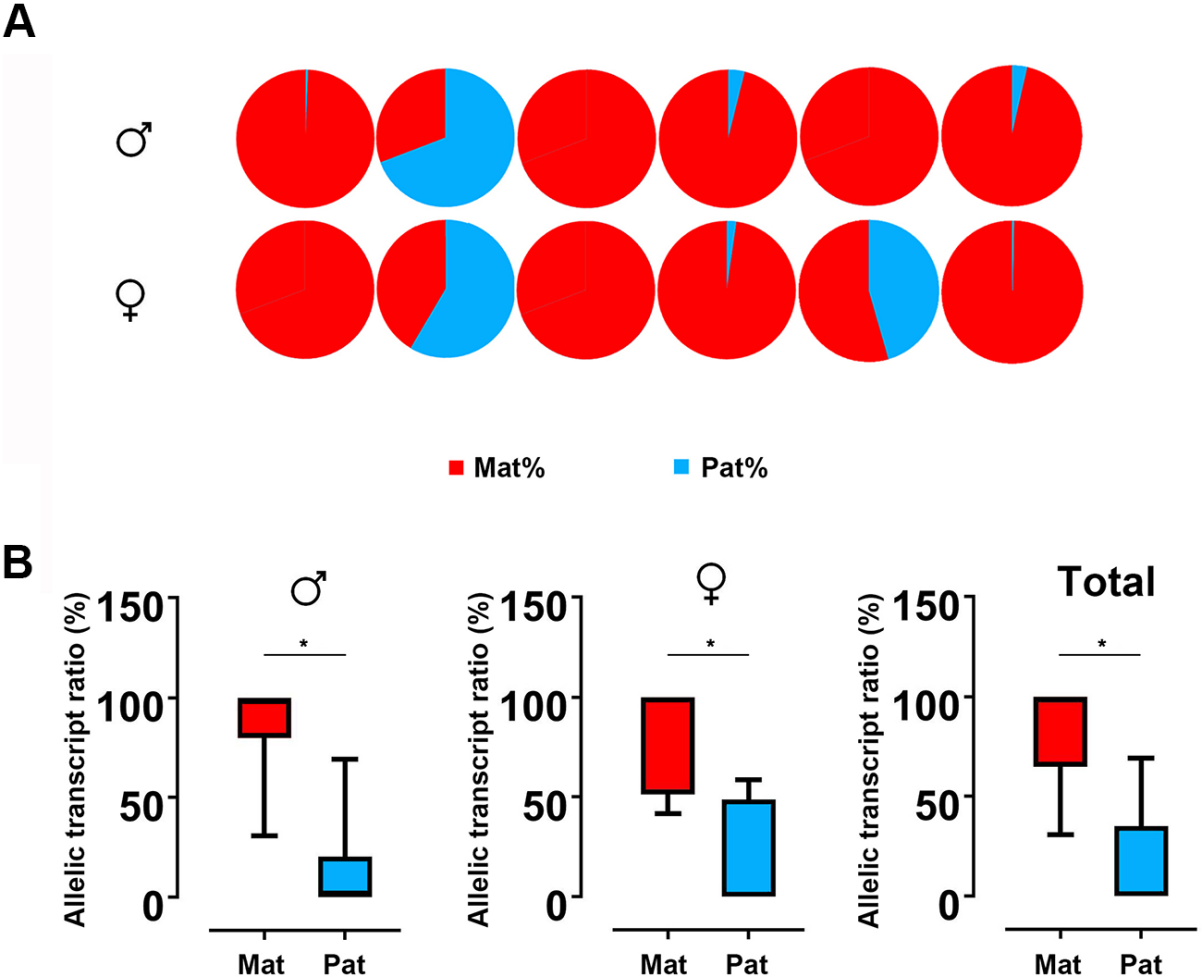
ASE analyses of GH in hybrid. **(A)** Asymmetric allelic expression of GH in male hybrids and female hybrids by pyrosequencing. Six individuals of male hybrids and six female hybrids were included based on the C/G SNP site (nucleotide 501). **(B)** Allelic transcript ratios of C/G in hybrid calculated from males (n = 6), females (n = 6) and total studied individuals (n = 12). Data are presented as the mean ± SEM. Asterisks indicate significant differences between groups (*P* < 0.05). Mat, maternal; Pat, parental.

Based on the SNP of nucleotide 501, we used pyrosequencing to measure GH ASE. The allelic expression was carried out for six males and six females in hybrids. The results showed that both males and females showed allelic expression imbalance. In males, five individuals were significantly dominated by maternal alleles, while only one individual was dominated by paternal allele. This result is similar to that observed in females, in which five in six individuals showed bias of maternal alleles (**Figure 4A**). By *t* test, a strong maternal bias was found both in males (maternal:paternal = 87.10:12.90) and females (maternal:paternal = 82.33:17.77) (**Figure 4B**).

**Figure 5:**
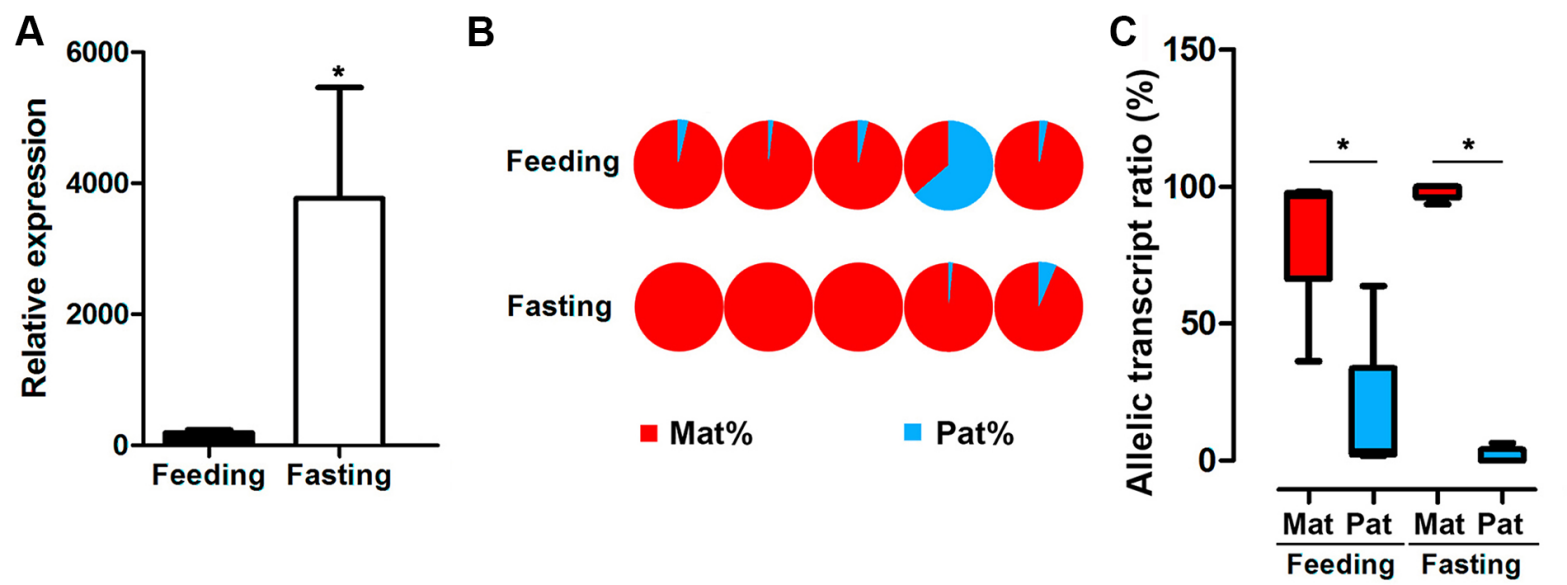
Expression of GH in hybrid tilapia in feeding and fasting conditions. **(A)** Increasing overall expression of GH in hybrid tilapia by RT-PCR after fasting in hybrid tilapia. Data are presented as the mean ± SEM (n = 4). Asterisks indicate significant differences between groups (*P* < 0.05). **(B)** ASE of GH in feeding and fasting hybrids (n = 5) by pyrosequencing based on the C/G SNP site (nucleotide 501). **(C)** Allelic transcript ratios of C/G in feeding and fasting hybrids. Data are presented as the mean ± SEM (n = 6). Asterisks indicate significant differences between groups (*P* < 0.05). Mat, maternal; Pat, parental.

GH expression is controlled by nutritional conditions, and male hybrids are major farmed population. Thus, we assayed allele-specific GH gene expression in male hybrids under feeding or fasting status. The results indicated that fasting significantly increased the mRNA expression of GH in the pituitary from the male hybrid compared to the feeding fish (**Figure 5A**). Nevertheless, a strong bias of maternal allele expression was found in both feeding and fasting hybrids. In the feeding group, four in five individuals were dominated by maternal alleles. All of the individuals had maternal bias by ASE analysis, showing allelic expression imbalance in the fasting group (**Figures 5B, C**).

### *Cis*- and *Trans*-Regulation of GH

The nonadditive expression and allelic expression imbalance were derived by the regulation of transcriptional activities from the subgenomes in hybrids. We further analyzed GH transcriptional regulation by identifying the *cis*- and *trans*-regulation of GH. Interspecies hybrid F_1_ could be used for distinguished *cis*- and *trans*-regulation (Shi et al., 2012). The *cis* and *trans* effect (we denote by A) could be determined by expression divergence between parents. In hybrids, allelic expression divergence between two subgenomes was used to determine the *cis* effect (we denote by B). Thus, the *trans* effect could be calculated by (A)-(B). We used the relative expression values of GH in Nile tilapia and blue tilapia to calculate (A) (**Figure 2B**) and ASE values from the hybrid to calculate (B) (**Figure 4B**). The results showed that GH was under *cis*- and *trans*-regulation, either in males or in females.

**Figure 6:**
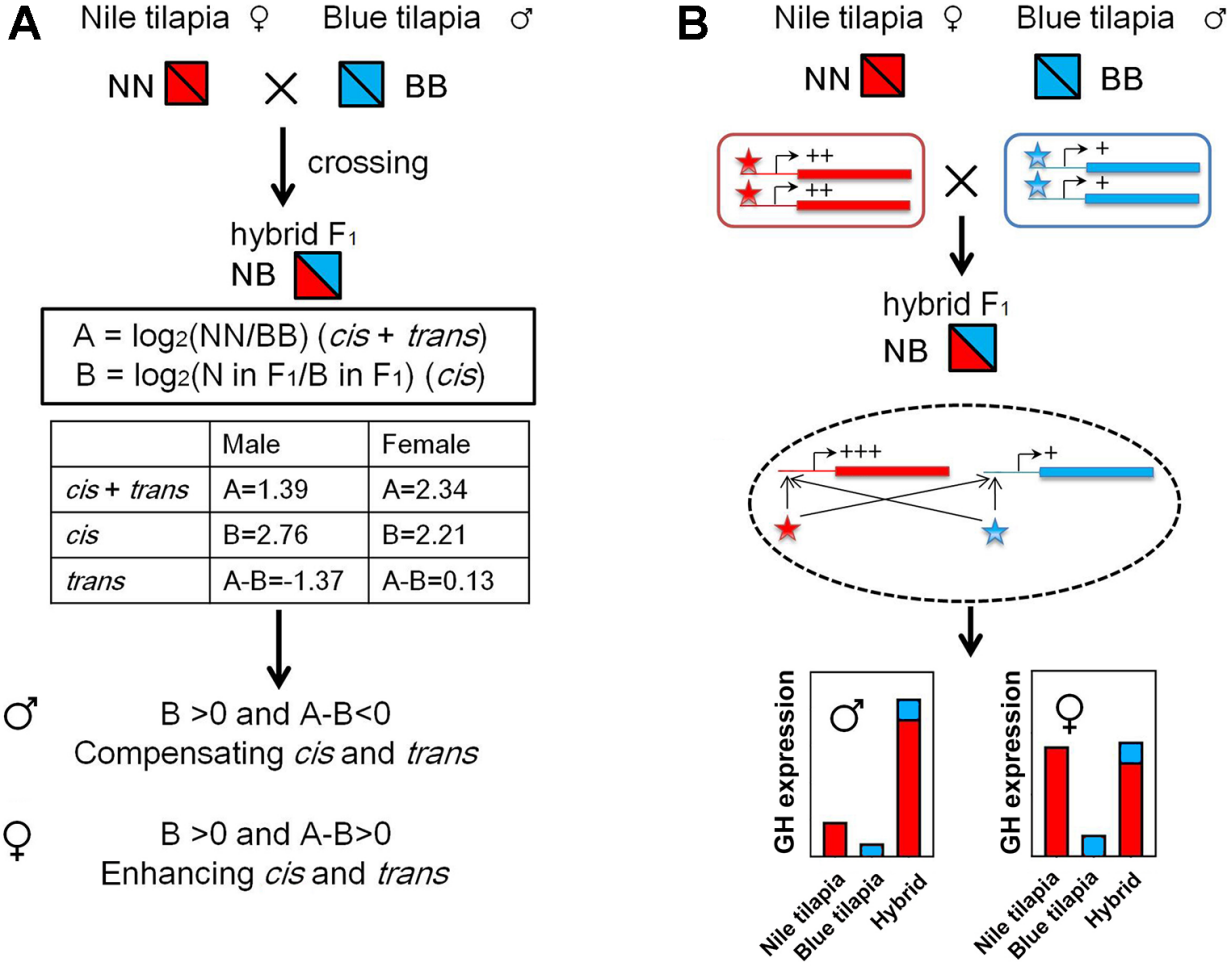
GH *cis* and *trans* regulation in hybrid tilapia. **(A)** Identification of enhancing *cis* and *trans* effects was found in the hybrid by calculating the value of overall expression (data from **Figure 2**) and ASE (data from **Figure 4**). **(B)** Possible mode of N and B allele interactions contributes to gene expression changes in the hybrid. In hybrids, both *cis* and *trans* effects tend to increase the transcriptional activities of GH genes in the same direction. These regulations were more sensitive for the subgenome of Nile tilapia. As a result, GH in the hybrid is higher than that in its parents, while the expressions of B alleles were not changed. Nile tilapia, blue tilapia, and hybrid were represented as NN, BB, and NB, respectively. Red and blue boxes indicate genes of Nile tilapia and blue tilapia, respectively. Lines attached to the genes indicate *cis* elements. Asterisks indicate *trans* regulators (Nile tilapia in red; blue tilapia in blue). +: low expression level; ++: medium expression level; +++: high expression level.

Furthermore, compensating *cis* and *trans* effects were found in the male hybrid by comparing the B and A-B values (B > 0 and A-B < 0). In contrast, the *cis* and *trans* effects act in the same direction as enhancing *cis* and *trans* effects in females, since B > 0 and A-B > 0 were found (**Figure 6A**). In hybrid tilapia, *cis* and *trans* regulators from the Nile tilapia subgenome have larger (or dominant) effects on allelic expression divergence than do those from blue tilapia (**Figure 6B**).

## Discussion

Heterosis, as a heavily researched subject in the evolution and breeding fields, has attracted considerable attention in recent years; however, the basic mechanisms of heterosis are still unclear, especially in vertebrates. In this study, by interspecies hybridization, we investigated the details of heterosis in fish. The hybrid of Nile tilapia (♀) × blue tilapia (♂) is a popular farmed tilapia line that has advantages of fast growth, strong resistance to disease, and low temperature resistance. The growth vigor in this hybrid is recognized compared to its original parents. In the present study, we first obtained a pure line of parents by nine rounds of self-breeding and then generated the hybrids for further investigation. Similar to previous results, the hybrids displayed growth vigor compared to their parents (Pruginin et al., 1975). This hybrid line provides a good model for studying successfully manipulated heterosis. In this study, we used GH as an indicator of the growth vigor formation in hybrid F_1_.

GH is a hormone that has been regarded as secreted by the pituitary dominantly (Inoue et al., 2003; Zhong et al., 2012). Our results also indicated that unique expression of GH was found in the pituitary of hybrid tilapia. Thus, the mRNA expression patterns were analyzed in the pituitary. The connection between fast growth appearance and high GH expression has been demonstrated previously. Both the domestication and GH transgenic coho salmon (*Oncorhynchus kisutch*) had higher GH expression levels than that in wild fish and exhibited fast growth appearance (Devlin et al., 2009). GH transgenic common carp (*Cyprinus carpio*) (Wu et al., 2003) and GH transgenic triploid fish showed a notable growth performance (Yu et al., 2011). In the present study, the results demonstrated higher expression of GH in the hybrid compared to its parents. In males, the GH in the hybrid was higher than that in both parents, which is “overdominant.” In females, GH in the hybrid had no significant difference compared to Nile tilapia but higher than that in blue tilapia, showing “maternal dominance.” This finding is in accordance with the growth vigor in hybrid tilapia compared with Nile tilapia and blue tilapia. Growth vigor generated by intercross hybridization has been used in fish breeding for several decades (Bartley et al., 2000). In hybrids, nonadditive expression could be used to illustrate the mechanism of hybrid vigor (Chen, 2013). We used MPV to determine the nonadditive expression of GH in hybrid tilapia. The results showed that GH mRNA levels were significantly higher in hybrids than MPV, indicating nonadditive expression of GH in hybrid tilapia; in other words, the superiority of GH expression is a major cause of growth vigor in hybrid.

After determining the GH expression superiority in hybrids, the next question to answer is how the transcription of the two subgenomes led to higher expression in hybrids. We extended the expression study to the ASE to answer the question. In hybrids, subgenome expression contributes to the mRNA levels, while asymmetric allelic expressions are common features in hybrids. ASE is crucial for mammalian development. For instance, IGF-2, as a famous imprinted gene, participates in embryo development (Reik et al., 2004). Loss of the IGF-2 imprinting results in overgrowth of fetal tissue and may lead to death (Lau et al., 1994). Intriguingly, the present data showed that GH had maternal allelic bias in hybrid tilapia. All-female Amazon molly was generated by distant hybridization of the Atlantic molly (*Poecilia mexicana*) and the sailfin molly (*Poecilia latipinna*). The ASE analysis of androgen receptor α suggested a maternal bias in ovary from Amazon molly (Zhu et al., 2017a). There were high ratios of maternal homolog expression of transcripts in diploid and tetraploid hybrids of *Carassius auratus* red var. (♀) and *C. carpio* (♂) (Ren et al., 2016). The growth-related genes in the two hybrids also showed maternal bias (Ren et al., 2016). Thus, maternal bias may be universal in hybrid descendants. In addition, we still noticed that one male and one female showed a paternal bias, which suggested that the ASE is not completely coincident in the F_1_ population. This finding may result from the unstable genome and/or pretranscriptional regulation in the newly formed hybrid.

The fasting and feeding effects on GH levels in hybrids were determined to illustrate the mRNA expression characteristics under different nutrient conditions. The results suggested that fasting treatment stimulated GH expression in the hybrid. In channel catfish (*Ictalurus punctatus*), 4 weeks of fasting increased GH mRNA expression significantly (Small and Peterson, 2005). Elevated GH mRNA levels were also found in fasting *Oreochromis mossambicus* (Fox et al., 2006). Still, the ASE of GH in fasting fish was a significant maternal origin. Thus, the fasting stimulation of GH is mainly achieved by inducing the transcriptional activity of the maternal genome. ASE was regulated by planting density and drought stress in maize hybrids (Guo et al., 2004). Allelic, additively expressed genes were regulated by the high-yielding environments in maize (Guo et al., 2006). In humans, the homocysteine concentrations affected monoallelic to biallelic expression in patients with uremia (Ingrosso et al., 2003). To the best of our knowledge, the present study is the first to show the GH ASE in hybrid tilapia with nutrient changes. The results suggested that nutrient conditions affect GH expression, but ASE is not changed by feeding or fasting.

In hybrid tilapia, it is observed that both *cis* and *trans* regulators of Nile tilapia (maternal) have larger (or more dominant) effects than those of blue tilapia (paternal) on allelic expression divergence. However, different regulations of *cis* and *trans* effects were observed in males and females. Compensating *cis* and *trans* effects in males and enhancing *cis* and *trans* effects in females were observed, respectively. As reported in other hybrids (Landry et al., 2005; Goncalves et al., 2012), compensating *cis* and *trans* effects were more common than enhancing *cis* and *trans* effects, which can cause underdominant or overdominant gene expression. Indeed, our present results also suggested that male hybrid tilapia exhibited overdominant GH expression. In contrast, enhancing *cis* and *trans* effects in females could increase the levels of gene-expression diversity, as in plants (Shi et al., 2012). It is noteworthy that the direction of *cis* and *trans* effects may differ depending on sex in further studies. Based on this evidence, we presumed that the hybrid tilapia with a high level of homozygosity had higher GH expression mediated by the interaction between *trans* factors and *cis* elements. Similarly, these effects were also found in genes associated with abiotic stimuli in plants with increasing gene expression (Shi et al., 2012). The *trans* factors expressed from Nile tilapia and blue tilapia strongly promoted GH expression from Nile tilapia, rather than blue tilapia. Meanwhile, the *cis* elements are responsible for the binding activities of the *trans* factors. In contrast, the subgenome of blue tilapia seems to be inhibited. Subsequently, these effects lead to higher GH expression with maternal dominance of the subgenome after hybridization.

Taken together, our findings support the superiority of GH expression that nonadditive expression of GH is related to growth vigor in hybrid tilapia. The maternal bias of GH ASE was found and does not rely on sex or nutritional conditions. These expression characteristics may be mediated by compensating *cis* and *trans* effects in males and enhancing *cis* and *trans* effects in females, respectively. Epigenetic regulatory mechanisms, such as DNA methylation and noncoding RNA, may be superimposed on *cis* and *trans* regulation.

## Data Availability Statement

The data supporting the conclusions of this study can be found in the EVA under accession number PRJEB34193 (https://www.ebi.ac.uk/ena/data/view/PRJEB34193). 

## Ethics Statement

The animal study was reviewed and approved by Animal Research and Ethics Committees of Guangxi Academy of Fishery Sciences.

## Author Contributions

HZ, XZ, QX, JY, and ZH carried out the experiments. HZ and YZ designed the experiment and wrote the article. HFZ, JX, ZT, FW, and YL interpreted the results and revised the article. All authors read and approved the manuscript.

## Funding

The work was supported by the National Natural Science Foundation of China [31672627, 31460688, 31760756, 31660736], National Key Research and Development Program of China [2018YFD0900601], the Natural Science Foundation of Guangxi [2017GXNSFFA198001], and the China Agriculture Research System [CARS-46].

## Conflict of Interest

The authors declare that the research was conducted in the absence of any commercial or financial relationships that could be construed as a potential conflict of interest.
