# Supplementary material for: Nonadditive and Asymmetric Allelic Expression of Growth Hormone in Hybrid Tilapia
Source: Front Genet. 2019 Oct 15;10:961. doi: 10.3389/fgene.2019.00961 (PMC6803431; doi:10.3389/fgene.2019.00961)
Supplement: Supplementary file 1 [file Table_1.docx]

Supplementary data 1

Four SNPs were identified from the cDNA sequences of Nile tilapia and blue tilapia. (A) Fasta data of cDNA sequences of Nile tilapia (N1 – N3) and blue tilapia (B1 – B3). (B) ClustalW analysis indicated four SNP sites (nucleotides 53, 327, 501 and 603) between Nile tilapia and blue tilapia in gene coding region. Red bold letters indicate the SNP sites.

**(A)**

>N1

TCTGAGCCGCAAACAGAGCCTGAACTGATGCCAGCCATGAACTCAGTCGTCCTCCTGCTGTCGGTTGTGTGTTTGGGCG**T**CTCCTCTCAGCAGATCACAGACAGCCAGCGTTTGTTCTCCATTGCAGTCAACAGAGTCACGCACCTGCACCTGCTCGCCCAGAGACTCTTCTCGGACTTTGAGAGCTCTCTGCAGACGGAGGAGCAACGTCAGCTCAACAAAATCTTCCTGCAGGACTTCTGCAACTCTGATTACATCATCAGCCCGATCGACAAACACGAGACGCAGCGCAGCTCGGTCCTGAAGCTGCTGTCGATCTCCTATGGACTGGTTGAGTCCTGGGAGTTTCCCAG**T**CGCTCTCTGTCTGGAGGTTCCTCTCTGAGGAACCAGATTTCACCAAGGCTGTCTGAGCTTAAAACGGGAATCTTGCTGCTGATCAGGGCCAATCAGGATGAAGCAGAGAATTATCCTGACACCGACACCCTCCAGCACGCTCCTTACGGAAACTATTATCAAAGTCTGGGAGG**C**AACGAATCGCTGAGACAAACTTATGAATTGCTGGCTTGCTTCAAGAAGGACATGCACAAGGTGGAGACCTACCTGACGGTAGCTAAATGTCGACTCTCTCC**A**GAAGCAAACTGCACTCTGTAG

>N2

TCTGAGCCGCAAACAGAGCCTGAACTGATGCCAGCCATGAACTCAGTCGTCCTCCTGCTGTCGGTTGTGTGTTTGGGCG**T**CTCCTCTCAGCAGATCACAGACAGCCAGCGTTTGTTCTCCATTGCAGTCAACAGAGTCACGCACCTGCACCTGCTCGCCCAGAGACTCTTCTCGGACTTTGAGAGCTCTCTGCAGACGGAGGAGCAACGTCAGCTCAACAAAATCTTCCTGCAGGACTTCTGCAACTCTGATTACATCATCAGCCCGATCGACAAACACGAGACGCAGCGCAGCTCGGTCCTGAAGCTGCTGTCGATCTCCTATGGACTGGTTGAGTCCTGGGAGTTTCCCAG**T**CGCCCTCTGTCTGGAGGTTCCTCTCTGAGGAACCAGATTTCACCAAGGCTGTCTGAGCTTAAAACGGGAATCTTGCTGCTGATCAGGGCCAATCAGGATGAAGCAGAGAATTATCCTGACACCGACACCCTCCAGCACGCTCCTTACGGAAACTATTATCAAAGTCTGGGAGG**C**GACGAATCGCTGAGACAAACTTATGAATTGCTGGCTTGCTTCAAGAAGGACATGCACAAGGTGGAGACCTACCTGACGGTAGCTAAATGTCGACTCTCTCC**A**GAAGCAAACTGCACTCTGTAG

>N3

TCTGAGCCGCAAACAGAGCCTGAACTGATGCCAGCCATGAACTCAGTCGTCCTCCTGCTGTCGGTTGTGTGTTTGGGCG**T**CTCCTCTCAGCAGATCACAGACAGCCAGCGTTTGTTCTCCATTGCAGTCAACAGAGTCACGCACCTGCACCTGCTCGCCCAGAGACTCTTCTCGGACTTTGAGAGCTCTCTGCAGACGGAGGAGCAACGTCAGCTCAACAAAATCTTCCTGCAGGACTTCTGCAACTCTGATTACATCATCAGCCCGATCGACAAACACGAGACGCAGCGCAGCTCGGTCCTGAAGCTGCTGTCGATCTCCTATGGACTGGTTGAGTCCTGGGAGTTTCCCAG**T**CGCTCTCTGTCTGGAGGTTCCTCTCTGAGGAACCAGATTTCACCAAGGCTGTCTGAGCTTAAAACGGGAATCTTGCTGCTGATCAGGGCCAATCAGGATGAAGTAGAGAATTATCCTGACACCGACACCCTCCAGCACGCTCCTTACGGAAACTATTATCAAAGTCTGGGAGG**C**AACGAATCGCTGAGACAAACTTATGAATTGCTGGCTTGCTTCAAGAAGGACATGCACAAGGTGGAGACCTACCTGACGGTAGCTAAATGTCGACTCTCTCC**A**GAAGCAAACTGCACTCTGTAG

>B1

TCTGAGCCGCAAACAGAGCCTGAACTGATGCCAGCCATGAACTCAGTCGTCCTCCTGCTGTCGGTTGTGTGTTTGGGCG**C**CTCCTCTCAGCAGATCACAGACAGCCAGCGTTTGTTCTCCATTGCAGTCAACAGAGTCACGCACCTGCACCTGCTCGCCCAGAGACTCTTCTCGGACTTTGAGAGCTCTCTGCAGACGGAGGAGCAACGTCAGCTCAACAAAATCTTCCTGCAGGACTTCTGCAACTCTGATTACATCATCAGCCCGATCGACAAACACGAGACGCAGCGCAGCTCGGTCCTGAAGCTGCTGTCGATCTCCTATGGACTGGTTGAGTCCTGGGAGTTTCCCAG**C**CGCTCTCTGTCTGGAGGTTCCTCTCTGAGGAACCAGATTTCACCAAGGCTGTCTGAGCTTAAAACGGGAATCTTGCTGCTGATCAGGGCCAATCAGGATGAAGCAGAGAATTATCCTGACACCGACACCCTCCAGCACGCTCCTTACGGAAACTATTATCAAAGTCTGGGAGG**G**AACGAATCGCTGAGACAAACTTATGAATTGCTGGCTTGCTTCAAGAAGGACATGCACAAGGTGGAGACCTACCTGACGGTAGCTAAATGTCGACTCTCTCC**G**GAAGCAAACTGCACTCTGTAG

>B2

TCTGAGCCGCAAACAGAGCCTGAACTGATGCCAGCCATGAACTCAGTCGTCCTCCTGCTGTCGGTTGTGTGTTTGGGCG**C**CTCCTCTCAGCAGATCACAGACAGCCAGCGTTTGTTCTCCATTGCAGTCAACAGAGTCACGCACCTGCACCTGCTCGCCCAGAGACTCTTCTCGGACTTTGAGAGCTCTCTGCAGACGGAGGAGCAACGTCAGCTCAACAAAATCTTCCTGCAGGACTTCTGCAACTCTGATTACATCATCAGCCCGATCGACAAACACGAGACGCAGCGCAGCTCGGTCCTGAAGCTGCTGTCGATCTCCTATGGACTGGTTGAGTCCTGGGAGTTTCCCAG**C**CGCTCTCTGTCTGGAGGTTCCTCTCTGAGGAACCAGATTTCACCAAGGCTGTCTGAGCTTAAAACGGGAATCTTGCTGCTGATCAGGGCCAATCAGGATGAAGCAGAGAATTATCCTGACACCGACACCCTCCAGCACGCTCCTTACGGAAACTATTATCAAAGTCTGGGAGG**G**AACGAATCGCTGAGACAAACTTATGAATTGCTGGCTTGCTTCAAGAAGGACATGCACAAGGTGGAGACCTACCTGACGGTAGCTAAATGTCGACTCTCTCC**G**GAAGCAAACTGCACTCTGTAG

>B3

TCTGAGCCGCAAACAGAGCCTGAACTGATGCCAGCCATGAACTCAGTCGTCCTCCTGCTGTCGGTTGTGTGTTTGGGCG**C**CTCCTCTCAGCAGATCACAGACAGCCAGCGTTTGTTCTCCATTGCAGTCAACAGAGTCACGCACCTGCACCTGCTCGCCCAGAGACTCTTCTCGGACTTTGAGAGCTCTCTGCAGACGGAGGAGCAACGTCAGCTCAACAAAATCTTCCTGCAGGACTTCTGCAACTCTGATTACATCATCAGCCCGATCGACAAACACGAGACGCAGCGCAGCTCGGTCCTGAAGCTGCTGTCGATCTCCTATGGACTGGTTGAGTCCTGGGAGTTTCCCAG**C**CGCTCTCTGTCTGGAGGTTCCTCTCTGAGGAACCAGATTTCACCAAGGCTGTCTGAGCTTAAAACGGGAATCTTGCTGCTGATCAGGGCCAATCAGGATGAAGCAGAGAATTATCCTGACACCGACACCCTCCAGCACGCTCCTTACGGAAACTATTATCAAAGTCTGGGAGG**G**AACGAATCGCTGAGACAAACTTATGAATTGCTGGCTTGCTTCAAGAAGGACATGCACAAGGTGGAGACCTACCTGACGGTAGCTAAATGTCGACTCTCTCC**G**GAAGCAAACTGCACTCTGTAG

**(B)**

N2 TCTGAGCCGCAAACAGAGCCTGAACTGATGCCAGCCATGAACTCAGTCGTCCTCCTGCTG

N1 TCTGAGCCGCAAACAGAGCCTGAACTGATGCCAGCCATGAACTCAGTCGTCCTCCTGCTG

N3 TCTGAGCCGCAAACAGAGCCTGAACTGATGCCAGCCATGAACTCAGTCGTCCTCCTGCTG

B1 TCTGAGCCGCAAACAGAGCCTGAACTGATGCCAGCCATGAACTCAGTCGTCCTCCTGCTG

B2 TCTGAGCCGCAAACAGAGCCTGAACTGATGCCAGCCATGAACTCAGTCGTCCTCCTGCTG

B3 TCTGAGCCGCAAACAGAGCCTGAACTGATGCCAGCCATGAACTCAGTCGTCCTCCTGCTG

************************************************************

N2 TCGGTTGTGTGTTTGGGCG**T**CTCCTCTCAGCAGATCACAGACAGCCAGCGTTTGTTCTCC

N1 TCGGTTGTGTGTTTGGGCG**T**CTCCTCTCAGCAGATCACAGACAGCCAGCGTTTGTTCTCC

N3 TCGGTTGTGTGTTTGGGCG**T**CTCCTCTCAGCAGATCACAGACAGCCAGCGTTTGTTCTCC

B1 TCGGTTGTGTGTTTGGGCG**C**CTCCTCTCAGCAGATCACAGACAGCCAGCGTTTGTTCTCC

B2 TCGGTTGTGTGTTTGGGCG**C**CTCCTCTCAGCAGATCACAGACAGCCAGCGTTTGTTCTCC

B3 TCGGTTGTGTGTTTGGGCG**C**CTCCTCTCAGCAGATCACAGACAGCCAGCGTTTGTTCTCC

******************* ****************************************

N2 ATTGCAGTCAACAGAGTCACGCACCTGCACCTGCTCGCCCAGAGACTCTTCTCGGACTTT

N1 ATTGCAGTCAACAGAGTCACGCACCTGCACCTGCTCGCCCAGAGACTCTTCTCGGACTTT

N3 ATTGCAGTCAACAGAGTCACGCACCTGCACCTGCTCGCCCAGAGACTCTTCTCGGACTTT

B1 ATTGCAGTCAACAGAGTCACGCACCTGCACCTGCTCGCCCAGAGACTCTTCTCGGACTTT

B2 ATTGCAGTCAACAGAGTCACGCACCTGCACCTGCTCGCCCAGAGACTCTTCTCGGACTTT

B3 ATTGCAGTCAACAGAGTCACGCACCTGCACCTGCTCGCCCAGAGACTCTTCTCGGACTTT

************************************************************

N2 GAGAGCTCTCTGCAGACGGAGGAGCAACGTCAGCTCAACAAAATCTTCCTGCAGGACTTC

N1 GAGAGCTCTCTGCAGACGGAGGAGCAACGTCAGCTCAACAAAATCTTCCTGCAGGACTTC

N3 GAGAGCTCTCTGCAGACGGAGGAGCAACGTCAGCTCAACAAAATCTTCCTGCAGGACTTC

B1 GAGAGCTCTCTGCAGACGGAGGAGCAACGTCAGCTCAACAAAATCTTCCTGCAGGACTTC

B2 GAGAGCTCTCTGCAGACGGAGGAGCAACGTCAGCTCAACAAAATCTTCCTGCAGGACTTC

B3 GAGAGCTCTCTGCAGACGGAGGAGCAACGTCAGCTCAACAAAATCTTCCTGCAGGACTTC

************************************************************

N2 TGCAACTCTGATTACATCATCAGCCCGATCGACAAACACGAGACGCAGCGCAGCTCGGTC

N1 TGCAACTCTGATTACATCATCAGCCCGATCGACAAACACGAGACGCAGCGCAGCTCGGTC

N3 TGCAACTCTGATTACATCATCAGCCCGATCGACAAACACGAGACGCAGCGCAGCTCGGTC

B1 TGCAACTCTGATTACATCATCAGCCCGATCGACAAACACGAGACGCAGCGCAGCTCGGTC

B2 TGCAACTCTGATTACATCATCAGCCCGATCGACAAACACGAGACGCAGCGCAGCTCGGTC

B3 TGCAACTCTGATTACATCATCAGCCCGATCGACAAACACGAGACGCAGCGCAGCTCGGTC

************************************************************

N2 CTGAAGCTGCTGTCGATCTCCTATGGACTGGTTGAGTCCTGGGAGTTTCCCAG**T**CGCCCT

N1 CTGAAGCTGCTGTCGATCTCCTATGGACTGGTTGAGTCCTGGGAGTTTCCCAG**T**CGCTCT

N3 CTGAAGCTGCTGTCGATCTCCTATGGACTGGTTGAGTCCTGGGAGTTTCCCAG**T**CGCTCT

B1 CTGAAGCTGCTGTCGATCTCCTATGGACTGGTTGAGTCCTGGGAGTTTCCCAG**C**CGCTCT

B2 CTGAAGCTGCTGTCGATCTCCTATGGACTGGTTGAGTCCTGGGAGTTTCCCAG**C**CGCTCT

B3 CTGAAGCTGCTGTCGATCTCCTATGGACTGGTTGAGTCCTGGGAGTTTCCCAG**C**CGCTCT

***************************************************** *** **

N2 CTGTCTGGAGGTTCCTCTCTGAGGAACCAGATTTCACCAAGGCTGTCTGAGCTTAAAACG

N1 CTGTCTGGAGGTTCCTCTCTGAGGAACCAGATTTCACCAAGGCTGTCTGAGCTTAAAACG

N3 CTGTCTGGAGGTTCCTCTCTGAGGAACCAGATTTCACCAAGGCTGTCTGAGCTTAAAACG

B1 CTGTCTGGAGGTTCCTCTCTGAGGAACCAGATTTCACCAAGGCTGTCTGAGCTTAAAACG

B2 CTGTCTGGAGGTTCCTCTCTGAGGAACCAGATTTCACCAAGGCTGTCTGAGCTTAAAACG

B3 CTGTCTGGAGGTTCCTCTCTGAGGAACCAGATTTCACCAAGGCTGTCTGAGCTTAAAACG

************************************************************

N2 GGAATCTTGCTGCTGATCAGGGCCAATCAGGATGAAGCAGAGAATTATCCTGACACCGAC

N1 GGAATCTTGCTGCTGATCAGGGCCAATCAGGATGAAGCAGAGAATTATCCTGACACCGAC

N3 GGAATCTTGCTGCTGATCAGGGCCAATCAGGATGAAGTAGAGAATTATCCTGACACCGAC

B1 GGAATCTTGCTGCTGATCAGGGCCAATCAGGATGAAGCAGAGAATTATCCTGACACCGAC

B2 GGAATCTTGCTGCTGATCAGGGCCAATCAGGATGAAGCAGAGAATTATCCTGACACCGAC

B3 GGAATCTTGCTGCTGATCAGGGCCAATCAGGATGAAGCAGAGAATTATCCTGACACCGAC

************************************* **********************

N2 ACCCTCCAGCACGCTCCTTACGGAAACTATTATCAAAGTCTGGGAGG**C**GACGAATCGCTG

N1 ACCCTCCAGCACGCTCCTTACGGAAACTATTATCAAAGTCTGGGAGG**C**AACGAATCGCTG

N3 ACCCTCCAGCACGCTCCTTACGGAAACTATTATCAAAGTCTGGGAGG**C**AACGAATCGCTG

B1 ACCCTCCAGCACGCTCCTTACGGAAACTATTATCAAAGTCTGGGAGG**G**AACGAATCGCTG

B2 ACCCTCCAGCACGCTCCTTACGGAAACTATTATCAAAGTCTGGGAGG**G**AACGAATCGCTG

B3 ACCCTCCAGCACGCTCCTTACGGAAACTATTATCAAAGTCTGGGAGG**G**AACGAATCGCTG

*********************************************** ***********

N2 AGACAAACTTATGAATTGCTGGCTTGCTTCAAGAAGGACATGCACAAGGTGGAGACCTAC

N1 AGACAAACTTATGAATTGCTGGCTTGCTTCAAGAAGGACATGCACAAGGTGGAGACCTAC

N3 AGACAAACTTATGAATTGCTGGCTTGCTTCAAGAAGGACATGCACAAGGTGGAGACCTAC

B1 AGACAAACTTATGAATTGCTGGCTTGCTTCAAGAAGGACATGCACAAGGTGGAGACCTAC

B2 AGACAAACTTATGAATTGCTGGCTTGCTTCAAGAAGGACATGCACAAGGTGGAGACCTAC

B3 AGACAAACTTATGAATTGCTGGCTTGCTTCAAGAAGGACATGCACAAGGTGGAGACCTAC

************************************************************

N2 CTGACGGTAGCTAAATGTCGACTCTCTCC**A**GAAGCAAACTGCACTCTGTAG

N1 CTGACGGTAGCTAAATGTCGACTCTCTCC**A**GAAGCAAACTGCACTCTGTAG

N3 CTGACGGTAGCTAAATGTCGACTCTCTCC**A**GAAGCAAACTGCACTCTGTAG

B1 CTGACGGTAGCTAAATGTCGACTCTCTCC**G**GAAGCAAACTGCACTCTGTAG

B2 CTGACGGTAGCTAAATGTCGACTCTCTCC**G**GAAGCAAACTGCACTCTGTAG

B3 CTGACGGTAGCTAAATGTCGACTCTCTCC**G**GAAGCAAACTGCACTCTGTAG

***************************** *********************

Supplementary data 2

The third SNP site (nucleotide 501) identification by Sanger sequencing using genome DNA of Nile tilapia (N1 - N6), blue tilapia (B1 - B6) and hybrid (NB1 - NB6) showing in fasta data. Red bold letters indicate the third SNP sites (nucleotide 501).

>N1

CGCTCCTTACGGAAACTATTATCAAAGTCTGGGAGG**C**AACGAATCGCTGAGACAAACTTATGAATTGCTGGCTTGCTTCAAGAAGGACATGCACAAGGTGAGGTAGTGGATAATGGTGATGTCACTGTGATGATGACAATGATGTAATGATGGTGAAGATGACATTTTTGTTGCAGGTGGAGACCTACCTGACGGTAGCTAAATGTCGACTCTCTCCAGAAGCAAACTGCACTCTGTAGCTCCACCTAATATTGATACTGATACGTGCTCTGTAGCCCCACCCTCATGTTGGCAAACTCTGCTTACATGTGTTAGCAATAGCCAAAAGGAATAAAAAGGACCCGG

>N2

CGCTCCTTACGGAAACTATTATCAAAGTCTGGGAGG**C**AACGAATCGCTGAGACAAACTTATGAATTGCTGGCTTGCTTCAAGAAGGACATGCACAAGGTGAGGTAGTGGATAATGGTGATGTCACTGTGATGATGACAATGATGTAATGATGGTGAAGATGACATTTTTGTTGCAGGTGGAGACCTACCTGACGGTAGCTAAATGTCGACTCTCTCCAGAAGCAAACTGCACTCTGTAGCTCCACCTAATATTGATACTGATACGTGCTCTGTAGCCCCACCCTCATGTTGGCAAACTCTGCTTACATGTGTTAGCATAGCAATAGGAAAAAAAAACGGGA

>N3

CGCTCCTTACGGAAACTATTATCAAAGTCTGGGAGG**C**AACGAATCGCTGAGACAAACTTATGAATTGCTGGCTTGCTTCAAGAAGGACATGCACAAGGTGAGGTAGTGGATAATGGTGATGTCACTGTGATGATGACAATGATGTAATGATGGTGAAGATGACATTTTTGTTGCAGGTGGAGACCTACCTGACGGTAGCTAAATGTCGACTCTCTCCAGAAGCAAACTGCACTCTGTAGCTCCACCTAATATTGATACTGATACGTGCTCTGTAGCCCCACCCTCATGTTGGCAAACTCTGCTTACATGTGTTAGCATAGCAAATAGGGATAAAAAGACGGG

>N4

CGCTCCTTACGGAAACTATTATCAAAGTCTGGGAGG**C**AACGAATCGCTGAGACAAACTTATGAATTGCTGGCTTGCTTCAAGAAGGACATGCACAAGGTGAGGTAGTGGATAATGGTGATGTCACTGTGATGATGACAATGATGTAATGATGGTGAAGATGACATTTTTGTTGCAGGTGGAGACCTACCTGACGGTAGCTAAATGTCGACTCTCTCCAGAAGCAAACTGCACTCTGTAGCTCCACCTAATATTGATACTGATACGTGCTCTGTAGCCCCACCCTCATGTTGGCAAACTCTGCTTACATGTGTTAGCATAGCAAATAAGGAAAAAAAAGGCCGG

>N5

CGCTCCTTACGGAAACTATTATCAAAGTCTGGGAGG**C**AACGAATCGCTGAGACAAACTTATGAATTGCTGGCTTGCTTCAAGAAGGACATGCACAAGGTGAGGTAGTGGATAATGGTGATGTCACTGTGATGATGACAATGATGTAATGATGGTGAAGATGACATTTTTGTTGCAGGTGGAGACCTACCTGACGGTAGCTAAATGTCGACTCTCTCCAGAAGCAAACTGCACTCTGTAGCTCCACCTAATATTGATACTGATACGTGCTCTGTAGCCCCACCCTCATGTTGGCAAACTCTGCTTACATGTGTTAGCATAGCAATAGATAAAAAAGCCGT

>N6

CGCTCCTTACGGAAACTATTATCAAAGTCTGGGAGG**C**AACGAATCGCTGAGACAAACTTATGAATTGCTGGCTTGCTTCAAGAAGGACATGCACAAGGTGAGGTAGTGGATAATGGTGATGTCACTGTGATGATGACAATGATGTAATGATGGTGAAGATGACATTTTTGTTGCAGGTGGAGACCTACCTGACGGTAGCTAAATGTCGACTCTCTCCAGAAGCAAACTGCACTCTGTAGCTCCACCTAATATTGATACTGATACGTGCTCTGTAGCCCCACCCTCATGTTGGCAAACTCTGCTTACATGTGTTAGCATTAGCAATAGGATAATAATGGCCCGGC

>B1

CGCTCCTTACGGAAACTATTATCAAAGTCTGGGAGG**G**AACGAATCGCTGAGACAAACTTATGAATTGCTGGCTTGCTTCAAGAAGGACATGCACAAGGTGAGGTAGTGGATAATGGTGATGTCACTGTGATGATGACAATGATGTAATGATGGTGAAGATGACATTTTTGTTGCAGGTGGAGACCTACCTGACGGTAGCTAAATGTCGACTCTCTCCGGAAGCAAACTGCACTCTGTAGCTCCACCTAATATTGATATTGATACGTGCTCTGTAGCCCCACCCTCATGTTGGCAAACTCTGCTTACATGTGTTAGCATTAGCAATAGGAAAAAAGCCGGG

>B2

CGCTCCTTACGGAAACTATTATCAAAGTCTGGGAGG**G**AACGAATCGCTGAGACAAACTTATGAATTGCTGGCTTGCTTCAAGAAGGACATGCACAAGGTGAGGTAGTGGATAATGGTGATGTCACTGTGATGATGACAATGATGTAATGATGGTGAAGATGACATTTTTGTTGCAGGTGGAGACCTACCTGACGGTAGCTAAATGTCGACTCTCTCCGGAAGCAAACTGCACTCTGTAGCTCCACCTAATATTGATATTGATACGTGCTCTGTAGCCCCACCCTCATGTTGGCAAACTCTGCTTACATGTGTTAGCATAGGCAAATAGGAATAATATAAAACGGGG

>B3

CGCTCCTTACGGAAACTATTATCAAAGTCTGGGAGG**G**AACGAATCGCTGAGACAAACTTATGAATTGCTGGCTTGTTTCAAGAAGGACATGCACAAGGTGAGGTAGTGGATAATGGTGATGTCACTGTGATGATGACAATGATGTAATGATGGTGAAGATGACATTTTTGTTGCAGGTGGAGACCTACCTGACGGTAGCTAAATGTCGACTCTCTCCGGAAGCAAACTGCACTCTGTAGCTCCACCTAATATTGATATTGATACGTGCTCTGTAGCCCCACCCTCATGTTGGCAAACTCTGCTTACATGTGTTAGCATAGCCAATAGGATAATAAGGCCCCGG

>B4

CGCTCCTTACGGAAACTATTATCAAAGTCTGGGAGG**G**AACGAATCGCTGAGACAAACTTATGAATTGCTGGCTTGCTTCAAGAAGGACATGCACAAGGTGAGGTAGTGGATAATGGTGATGTCACTGTGATGATGACAATGATGTAATGATGGTGAAGATGACATTTTTGTTGCAGGTGGAGACCTACCTGACGGTAGCTAAATGTCGACTCTCTCCGGAAGCAAACTGCACTCTGTAGCTCCACCTAATATTGATATTGATACGTGCTCTGTAGCCCCACCCTCATGTTGGCAAACTCTGCTTACATGTGTTAGCATAGCAATAGATATTTGAACCG

>B5

CGCTCCTTACGGAAACTATTATCAAAGTCTGGGAGG**G**AACGAATCGCTGAGACAAACTTATGAATTGCTGGCTTGCTTCAAGAAGGACATGCACAAGGTGAGGTAGTGGATAATGGTGATGTCACTGTGATGATGACAATGATGTAATGATGGTGAAGATGACATTTTTGTTGCAGGTGGAGACCTACCTGACGGTAGCTAAATGTCGACTCTCTCCGGAAGCAAACTGCACTCTGTAGCTCCACCTAATATTGATATTGATACGTGCTCTGTAGCCCCACCCTCATGTTGGCAAACTCTGCTTACATGTGTTAGCATTAGCAATAGGAATAT

>B6

CGCTCCTTACGGAAACTATTATCAAAGTCTGGGAGG**G**AACGAATCGCTGAGACAAACTTATGAATTGCTGGCTTGCTTCAAGAAGGACATGCACAAGGTGAGGTAGTGGATAATGGTGATGTCACTGTGATGATGACAATGATGTAATGATGGTGAAGATGACATTTTTGTTGCAGGTGGAGACCTACCTGACGGTAGCTAAATGTCGACTCTCTCCGGAAGCAAACTGCACTCTGTAGCTCCACCTAATATTGATATTGATACGTGCTCTGTAGCCCCACCCTCATGTTGGCAAACTCTGCTTACATGTGTTAGCATAGCAAATAGATAATAATGACCGGG

>NB1

CGCTCCTTACGGAAACTATTATCAAAGTCTGGGAGG**C/G**AACGAATCGCTGAGACAAACTTATGAATTGCTGGCTTGCTTCAAGAAGGACATGCACAAGGTGAGGTAGTGGATAATGGTGATGTCACTGTGATGATGACAATGATGTAATGATGGTGAAGATGACATTTTTGTTGCAGGTGGAGACCTACCTGACGGTAGCTAAATGTCGACTCTCTCCAGAAGCAAACTGCACTCTGTAGCTCCACCTAATATTGATATTGATACGTACTCTGTAGCCCCACCCTCATGTTGGCAAACTCTGCTTACATGTGTTAGCATAGCAATAGATATAATGTCG

>NB2

CGCTCCTTACGGAAACTATTATCAAAGTCTGGGAGG**C/G**AACGAATCGCTGAGACAAACTTATGAATTGCTGGCTTGCTTCAAGAAGGACATGCACAAGGTGAGGTAGTGGATAATGGTGATGTCACTGTGATGATGACAATGATGTAATGATGGTGAAGATGACATTTTTGTTGCAGGTGGAGACCTACCTGACGGTAGCTAAATGTCGACTCTCTCCGGAAGCAAACTGCACTCTGTAGCTCCACCTAATATTGATATTGATACGTGCTCTGTAGCCCCACCCTCATGTTGGCAAACTCTGCTTACATGTGTTAGCATAGCAATAGGATAATTGACGG

>NB3

CGCTCCTTACGGAAACTATTATCAAAGTCTGGGAGG**C/G**AACGAATCGCTGAGACAAACTTATGAATTGCTGGCTTGCTTCAAGAAGGACATGCACAAGGTGAGGTAGTGGATAATGGTGATGTCACTGTGATGATGACAATGATGTAATGATGGTGAAGATGACATTTTTGTTGCAGGTGGAGACCTACCTGACGGTAGCTAAATGTCGACTCTCTCCGGAAGCAAACTGCACTCTGTAGCTCCACCTAATATTGATATTGATACGTACTCTGTAGCCCCACCCTCATGTTGGCAAACTCTGCTTACATGTGTTAGCATTAGCAATAGGAAAAC

>NB4

CGCTCCTTACGGAAACTATTATCAAAGTCTGGGAGG**C/G**AACGAATCGCTGAGACAAACTTATGAATTGCTGGCTTGCTTCAAGAAGGACATGCACAAGGTGAGGTAGTGGATAATGGTGATGTCACTGTGATGATGACAATGATGTAATGATGGTGAAGATGACATTTTTGTTGCAGGTGGAGACCTACCTGACGGTAGCTAAATGTCGACTCTCTCCGGAAGCAAACTGCACTCTGTAGCTCCACCTAATATTGATATTGATACGTGCTCTGTAGCCCCACCCTCATGTTGGCAAACTCTGCTTACATGTGTTAGCATAGCAATAGGATAA

>NB5

CGCTCCTTACGGAAACTATTATCAAAGTCTGGGAGG**C/G**AACGAATCGCTGAGACAAACTTATGAATTGCTGGCTTGCTTCAAGAAGGACATGCACAAGGTGAGGTAGTGGATAATGGTGATGTCACTGTGATGATGACAATGATGTAATGATGGTGAAGATGACATTTTTGTTGCAGGTGGAGACCTACCTGACGGTAGCTAAATGTCGACTCTCTCCGGAAGCAAACTGCACTCTGTAGCTCCACCTAATATTGATATTGATACGTGCTCTGTAGCCCCACCCTCATGTTGGCAAACTCTGCTTACATGTGTTAGCATAGCAATAGAAAAA

>NB6

CGCTCCTTACGGAAACTATTATCAAAGTCTGGGAGG**C/G**AACGAATCGCTGAGACAAACTTATGAATTGCTGGCTTGCTTCAAGAAGGACATGCACAAGGTGAGGTAGTGGATAATGGTGATGTCACTGTGATGATGACAATGATGTAATGATGGTGAAGATGACATTTTTGTTGCAGGTGGAGACCTACCTGACGGTAGCTAAATGTCGACTCTCTCCGGAAGCAAACTGCACTCTGTAGCTCCACCTAATATTGATATTGATACGTACTCTGTAGCCCCACCCTCATGTTGGCAAACTCTGCTTACATGTGTTAGCATAGCAATAGGATAATATTGACGG
